# Associations of eHealth Literacy With Health Behavior Among Adult Internet Users

**DOI:** 10.2196/jmir.5413

**Published:** 2016-07-18

**Authors:** Seigo Mitsutake, Ai Shibata, Kaori Ishii, Koichiro Oka

**Affiliations:** ^1^ Research Team for Human Care Tokyo Metropolitan Institute of Gerontology Tokyo Japan; ^2^ Faculty of Health and Sport Sciences University of Tsukuba Ibaraki Japan; ^3^ Faculty of Sport Sciences Waseda University Saitama Japan

**Keywords:** health literacy, ehealth literacy, epatients, Internet, health behavior, cross-sectional studies

## Abstract

**Background:**

In the rapidly developing use of the Internet in society, eHealth literacy—having the skills to utilize health information on the Internet—has become an important prerequisite for promoting healthy behavior. However, little is known about whether eHealth literacy is associated with health behavior in a representative sample of adult Internet users.

**Objective:**

The aim of this study was to examine the association between eHealth literacy and general health behavior (cigarette smoking, physical exercise, alcohol consumption, sleeping hours, eating breakfast, eating between meals, and balanced nutrition) among adult Internet users in Japan.

**Methods:**

The participants were recruited among registrants of a Japanese Internet research service company and asked to answer a cross-sectional Internet-based survey in 2012. The potential respondents (N=10,178) were randomly and blindly invited via email from the registrants in accordance with the set sample size and other attributes. eHealth literacy was assessed using the Japanese version of the eHealth Literacy Scale. The self-reported health behaviors investigated included never smoking cigarettes, physical exercise, alcohol consumption, sleeping hours, eating breakfast, not eating between meals, and balanced nutrition. We obtained details of sociodemographic attributes (sex, age, marital status, educational attainment, and household income level) and frequency of conducting Internet searches. To determine the association of each health behavior with eHealth literacy, we performed a logistic regression analysis; we adjusted for sociodemographic attributes and frequency of Internet searching as well as for other health behaviors that were statistically significant with respect to eHealth literacy in univariate analyses.

**Results:**

We analyzed the data of 2115 adults (response rate: 24.04%, 2142/10,178; male: 49.74%, 1052/2115; age: mean 39.7, SD 10.9 years) who responded to the survey. Logistic regression analysis showed that individuals with high eHealth literacy were significantly more likely to exhibit the good health behaviors of physical exercise (adjusted odds ratio [AOR] 1.377, 95% CI 1.131-1.678) and eating a balanced diet (AOR 1.572, 95% CI 1.274-1.940) than individuals with low eHealth literacy.

**Conclusions:**

We found that some health behaviors, including exercise and balanced nutrition, were independently associated with eHealth literacy among Japanese adult Internet users.

## Introduction

According to an estimate of the Communications Usage Trend Survey in 2013, 82.8% of Japan’s general population are Internet users [1]. Approximately 70% of Japanese Internet users seek health information online [2]. One US study indicated that 72% of Internet users had looked online for health information over the previous year [3]; 59% of those who looked online for health information did so specifically to determine what medical condition they or an acquaintance might have [3]. In addition to improved medication compliance, decreased anxiety, and a greater feeling of safety, Internet users exhibit better self-care health behavior than those who do not use the Internet [4,5]. Thus, the Internet is increasingly becoming an effective information tool for attaining and maintaining better self-care health behavior [6,7].

In an information society, health literacy is growing in importance with respect to public health, and health care involves effectively using health information from multiple sources [8,9]. Health literacy—the degree to which individuals can obtain, process, and understand basic health information and services needed to make appropriate health decisions—is a key competence in promoting individual and public health [9]. Previous studies have identified an association between low health literacy and decreased knowledge of health care services and self-care management skills [8,10]. Toward improving health care quality and population health outcomes and achieving health equity, promoting health literacy is indicated as one of the objects of health communication and health information technology in “Healthy People 2020″ [11].

In this context, health information primarily relates to such electronic resources as the Internet and other technologies. Health information has notably assumed an important role in health promotion among the general public through the widespread use of personal computers and smartphones/mobile phones [6,7,12]. With this proliferation of online health information, one critical issue to have emerged is that many websites providing health information are invalid or difficult to understand; they may also be linked to commercial goods or private health services [13-15]. Regulating health information on the Internet is difficult because new information is constantly added. To utilize health information on the Internet properly, people seeking such information need to obtain “the ability to seek, find, understand, and appraise health information from electronic sources and apply the knowledge gained to addressing or solving a health problem” (ie, eHealth literacy) [15].

Previous studies of eHealth literacy have largely focused on defining the term [15-18], developing measures of eHealth literacy [2,19-23], and examining the effect of eHealth literacy interventions on people in need of it [24-27]. More recently, studies on eHealth literacy have examined the association between eHealth literacy and health-related outcomes. The Integrative Model of eHealth Use (IMeHU) suggests that social structures affect health behaviors through the microlevel conditions of eHealth literacy, motivation, and efficacy in using the Internet for health purposes [28]. Empirical studies have shown that individuals with high eHealth literacy had greater efficacy in finding health information and using health apps [29,30], were more active health information seekers [31-33], and employed more search strategies [33,34] than people with low eHealth literacy. Moreover, a limited number of studies have identified an association between eHealth literacy and health behaviors [35,36]. Hsu et al [36] showed that eHealth literacy mediated the association between individual factors and health behavior among college students; therefore, promoting health behavior among such students demands high levels of eHealth literacy. However, few studies have examined the relationship between eHealth literacy and health behavior in a general population.

The aim of the Healthy Japan 21 (second term) campaign of Japan’s Ministry of Health, Labour and Welfare is to prevent chronic diseases and improve daily health behavior with respect to smoking, exercise, alcohol, rest, and dietary habits among Japanese adults [37]. As in other developed countries, the Internet in Japan is a powerful means of promoting healthy behavior among college students as well as the adult population [1,2]. To design effective strategies for promoting healthy behavior among adult Internet users, it is necessary to examine the relationship between Internet use and such behavior. According to the IMeHU, eHealth literacy may play an important role in health behavior; however, little is known about the precise association in an adult population. Therefore, this study examines whether eHealth literacy is associated with various kinds of general health behavior: cigarette smoking, physical exercise, alcohol consumption, sleeping hours, eating breakfast, eating between meals, and balanced nutrition.

## Methods

### Participants

The study participants were recruited from the registrants of a Japanese Internet research service company called MyVoice Communication, Inc; the recipients were asked to respond to a cross-sectional Internet-based survey in 2012. In this study, we recruited individual Internet users because eHealth literacy is necessary to access online health information. We believed that an Internet survey would be appropriate for this study because responders to such a survey are clearly able to use the Internet effectively. The research company had approximately 1,180,000 voluntarily registered participants in 2012, and it obtained detailed sociodemographic data from each participant on registration. In this study, we aimed to collect data from 2000 men and women aged 20 to 59 years. We intended to minimize selection bias caused by proportional differences in terms of sex and age; therefore, we allocated the registered participants equally to eight sample groups categorized by sex and age (20-29, 30-39, 40-49, and 50-59 years), with n=250 in each group. The Internet research service company randomly chose the potential respondents from the registered participants in accordance with the sample sizes: N=10,178; male: 20-29 years, n=2275; 30-39 years, n=1255; 40-49 years, n=880; 50-59 years, n=699; female: 20-29 years, n=1979; 30-39 years, n=1362; 40-49 years, n=963; and 50-59 years, n=765. In addition, the Internet research service company blindly selected the potential respondents such that the authors and other registered participants were unable to identify those individuals.

The company invited registrants to participate in the survey by email. The number of potential respondents in each stratified sample group was determined by dividing the quota (n=250) by the response rate for the corresponding sociodemographic group. That response rate was computed based on the results of many previous surveys conducted by the research company. The questionnaires were placed in a protected area of a website, and the potential respondents received a specific URL in their invitation email. Potential respondents were able to log on to the protected area of the site using a unique ID and password. After the desired number of participants had voluntarily signed an online informed consent form and completed the sociodemographic data information form, further participants were no longer accepted. Reward points valued at 150 yen were provided as incentives for participation (US $1 was equivalent to approximately 82 yen in 2012). This study was approved by the Ethics Committee of Waseda University, Tokyo, Japan (No: 2011-245).

### Measurements

#### Sociodemographic Attributes

The research company provided categorized data as follows: sex (male, female); age group (20-29, 30-39, 40-49, and 50-59 years); marital status (not married, married); education level (up to high school, 2-year college or career college, college graduate or above); and household income level (<5 million yen, ≥5 million yen).

#### Frequency of Internet Searching

We assessed the frequency of information searches on the Internet in terms of daily conducted searches. We did so because one study found a positive association between eHealth literacy and the frequency of Internet searches [2]; we believed that the frequency of Internet searching could be used as a control variable for eHealth literacy and healthy behavior.

#### eHealth Literacy

We used the Japanese version of the eHealth Literacy Scale (eHEALS) to assess the eHealth literacy levels of participants [2]. The eHEALS consists of eight questions (see Multimedia Appendix 1); it uses a 5-point Likert scale, ranging from 1 (strongly disagree) to 5 (strongly agree), with a score range of 8 to 40 to measure the perceived eHealth literacy of participants [19]. The validity of the Japanese version of eHEALS (J-eHEALS) has been determined, and a confirmatory factor analysis using data from a previous survey [2] was conducted. This analysis for the 8-item model suggested a good fit for the proposed model (goodness-of-fit index=0.988, confirmatory fit index=0.993, root mean square error of approximation=0.056), and the internal reliability of the test was confirmed using Cronbach alpha coefficient (Cronbach alpha=.93) [2].

#### Health Behavior

Belloc and Breslow [38] have demonstrated the relationship between healthy behavior (including not smoking, regular physical exercise, moderate or no alcohol use, 7-8 hours’ sleep, eating breakfast, and not eating between meals) and positive health status. Based on the work of Belloc and Breslow, Hagihara and Morimoto [39] added balanced nutrition defined as eating meals with balanced nutrition to their list of healthy behavior. Many studies have referred to the work of Breslow and Enstrom [40] and Breslow and Breslow [41] with regard to health behavior, and Morimoto and associates [42,43] for health status; therefore, we followed the studies of Belloc and Breslow [38] and Hagihara and Morimoto [39] to ensure the survey quality. In accordance with previous studies about health behavior and status, in this study we used a self-administered questionnaire, which included items related to cigarette smoking, physical exercise, alcohol consumption, sleeping hours, eating breakfast, eating between meals, and balanced nutrition, to assess health behavior [38,39].

With respect to smoking status, the questionnaire included an item about whether participants had ever smoked. Physical exercise was assessed by asking participants about their weekly frequency. Alcohol consumption was determined by inquiring about the weekly frequency. Number of sleeping hours was evaluated in terms of daily sleeping hours. Eating breakfast and eating between meals were categorized as follows: every day, almost every day, sometimes, and never. Balanced nutrition was grouped into three categories: eating a nutritionally balanced diet, eating with little regard to nutritional balance, and not eating a balanced diet. In accordance with previous studies [38-43], each health behavior was divided into one of two categories (good health behavior; poor health behavior) as follows: smoking cigarettes (never smoking; smoking) [38,40,41], regular physical exercise (twice or more a week; less than once a week) [39,42,43], moderate or no alcohol use (less than four times a week; five or more times a week) [38-43], sleeping hours (7-8 hour per night; ≤6 or ≥9 hour per night) [38-43], eating breakfast (almost every day or every day; sometimes or never) [38-43], not eating between meals (sometimes or never; almost every day or every day) [38,40,41], and balanced nutrition (eating a nutritionally balanced diet; eating with little regard to diet or not eating a balanced diet) [39,42,43].

### Statistical Analyses

We divided J-eHEALS score into one of two categories (high or low) relative to the median group value (median 24.02, IQR 19.19-27.82); we did so in accordance with previous studies that used eHEALS to analyze the association between eHealth literacy level and health behavior and health information seeking [30,33,44]. We employed a chi-square test to evaluate the proportional differences in each health behavior with respect to eHealth literacy. We conducted logistic regression analyses to estimate the association between each health behavior and eHealth literacy level. To determine the association of each health behavior with eHealth literacy level, we performed logistic regression analyses: we adjusted for sociodemographic variables (age group, marital status, educational attainment, and household income), frequency of Internet searching, and health behaviors that were statistically significant with respect to eHealth literacy in univariate analyses. We calculated adjusted odds ratios and 95% confidence intervals for each variable. In all analyses, *P*<.05 was considered statistically significant. We used PASW 19.0 to compute the statistics.

## Results

### Sociodemographic Variables and Frequency of Internet Searching

We received the data for 2142 adults (response rate: 21.04%, 2142/10,178) from the research company. We excluded respondents with incomplete data (missing rate: 1.26%, 27/2142) and therefore analyzed the data of 2115 adults who provided complete information for the study variables. Table 1 presents the characteristics of the respondents. In this study, the mean age of the participants was 39.7 years (SD 10.9); 49.74% (1052/2115) of the participants were male, 50.69% (1072/2115) had graduated from college or graduate school, and 23.74% (502/2115) were educated to a level below a high school diploma. Among the respondents, 47.28% (1000/2115) had a household income less than 5 million yen and 52.72% (1115/2115) earned 5 million yen or more, 58.06% (1228/2115) were married, and 72.06% (1524/2115) used the Internet to search for information every day. The mean J-eHEALS score was 23.4 (SD 6.4).

**Table 1:** Sociodemographic characteristics of participants (N=2115).

Characteristics		n (%)
**Sex**		
	Male	1052 (49.74)
	Female	1063 (50.26)
**Age groups (years)**		
	20-29	527 (24.92)
	30-39	530 (25.06)
	40-49	531 (25.11)
	50-59	527 (24.92)
**Education level**		
	≤High school graduate	502 (23.74)
	Two-year college or career college	541 (25.58)
	≥College graduate	1072 (50.69)
**Household income (yen)**		
	<5 million	1000 (47.28)
	≥5 million	1115 (52.72)
**Marital status**		
	Not married	887 (41.94)
	Married	1228 (58.06)	
**Frequency of Internet searching (per week)**		
	Every day	1524 (72.06)
	No every day	591 (27.94)

### Association Between eHealth Literacy and Health Behavior

In the univariate analyses, sleeping hours (*P*=.30), eating breakfast (*P*=.75), and eating snacks (*P*=.17) were not statistically significantly related to eHealth literacy level. However, cigarette smoking (*P*<.001), physical exercise (*P*=.001), alcohol consumption (*P*=.009), and balanced nutrition (*P*<.001) were significantly related to eHealth literacy level; those variables were included in the logistic regression model as controlling factors. Table 2 presents the results of the logistic regression analysis for the association between eHealth literacy and different types of health behavior. This table also shows the results of the logistic regression analysis for the association between eHealth literacy and each type of health behavior after controlling for covariates. After controlling for covariates, individuals with high eHealth literacy were significantly more likely to exhibit good health behavior with respect to physical exercise (adjusted odds ratio [AOR] 1.377, 95% CI 1.131-1.678) and eating a balanced diet (AOR 1.572, 95% CI 1.274-1.940) than people with low eHealth literacy. However, after controlling for covariates, we observed no significant association between eHEALS score and health behavior with respect to cigarette smoking, alcohol consumption, sleeping hours, eating breakfast, and eating between meals.

**Table 2:** Association between eHealth literacy and health behavior.

Health behavior	OR (95% Cl)	*P*	AOR^a^ (95% Cl)	*P*
Cigarette smoking	0.866 (.729-1.029)	.10	0.862 (0.711-1.046)	.13
Physical exercise	1.470 (1.215-1.779)	<.001	1.377 (1.131-1.678)	.001
Alcohol consumption	0.847 (0.712-1.007)	.06	0.876 (0.727-1.055)	.88
Sleeping hours	1.039 (0.870-1.240)	.67	1.069 (0.890-1.282)	.48
Eating breakfast	1.198 (0.968-1.484)	.10	1.023 (0.814-1.286)	.84
Eating between meals	0.988 (0.829-1.177)	.89	1.044 (0.863-1.262)	.66
Balanced nutrition	1.764 (1.445-2.153)	<.001	1.572 (1.274-1.940)	<.001

^a^ Adjusted for sociodemographic factors, frequency of Internet searching, and other health behaviors that were statistically significant with respect to eHealth literacy in univariate analyses.

## Discussion

### Principal Results

After controlling for sociodemographic variables, frequency of Internet searching, and other health behavior, this study found that adult Internet users with high eHealth literacy were significantly more likely to have good health behavior, such as physical exercise and balanced nutrition, than individuals with low eHealth literacy. However, we found no significant association between eHealth literacy and cigarette smoking, alcohol consumption, sleeping hours, eating breakfast, or eating between meals.

### Comparison With Previous Work

This study is the first to examine the association between eHealth literacy and the general health behaviors of cigarette smoking, physical exercise, alcohol consumption, sleeping habits, eating breakfast, eating between meals, and balanced nutrition among Internet adult users in Japan. After controlling for covariates, we found eHealth literacy to be associated with the good health behavior of physical exercise and balanced nutrition among Internet users. The results of this study support those of the IMeHU [28]. According to the IMeHU, individuals with higher eHealth literacy had greater motivation and efficacy in using the Internet for health information [28]. Previous investigations have shown that individuals with high eHealth literacy were more active consumers of online health information [2,30,33,34,45]—especially information related to exercise and nutrition [2,45]—than people with low eHealth literacy. This study reinforces the IMeHU findings, whereby eHealth literacy may mediate the association between social status and health behavior through the use of online health information [28].

This study demonstrates that high eHealth literacy may promote the healthy behavior of physical exercise and balanced nutrition among the general population of Internet users. One study among college students found that eHealth literacy promotes such healthy behavior as exercising; eating low-fat foods, low-sugar cereals, and vegetables and fruit; and always having quality sleep [36]. This study expands on those findings by focusing not on college students, but the general population. Because approximately 90% of general adults aged 30 to 59 years have used the Internet, it is becoming an effective intervention tool for promoting health behavior among ordinary people [2]. Therefore, to promote healthy behavior, including physical exercise and balanced nutrition, it is necessary to examine ways of enhancing eHealth literacy among adult Internet users.

One study has found that functional eHealth literacy and critical eHealth literacy displayed a positive predictive power with respect to eating and exercise behavior, although critical eHealth literacy was able only to positively predict sleep behaviors [36]. Hsu et al [36] found that functional eHealth literacy and interactive eHealth literacy were less influential with respect to health behavior than critical eHealth literacy as follows: according to involvement theory, critical eHealth literacy may motivate individuals more to seek and evaluate the quality of health information than functional eHealth literacy and interactive eHealth literacy. In this study, however, eHEALS was used as a single factor, and it did not include the three dimensions of functional, interactive, and critical eHealth literacy [2,19]. Thus, this study does not allow any discussion of the association of health behavior with functional, interactive, and critical eHealth literacy. Further research is needed to clarify the mechanisms whereby the three dimensions of eHealth literacy affect health behavior toward developing an effective eHealth literacy educational program for promoting healthy behavior among adult Internet users.

We found that the mean eHEALS score among Japanese Internet users was lower than that previously reported in the United States [34]. Our finding is in line with that of a previous study on health literacy [46]. In this study, the mean eHEALS scores was 23.4 (SD 6.4), which is similar to that in a previous investigation of Japanese Internet users [35]. Conversely, Tennant et al [34] determined the mean eHEALS score to be 29.05 (SD 5.75) among baby boomers and older adults in the United States (male: 54.8%; age: mean 67.46, SD 9.98 years). Although the participants in this study were older than the population in our study, the mean eHEALS scores they reported were higher. This difference may be explained by the argument of Nakayama et al [46], whereby the Japanese population have found it difficult to find health information on the Internet because there is no reliable, understandable, neutral, and comprehensive health website comparable to websites such as MedlinePlus (US National Library of Medicine).

### Limitations

This study has a number of limitations. First, the participants were recruited from a single Japanese Internet research service company; thus, the relationships assessed may have been biased because of the potentially nonrepresentative nature of this sample as general Japanese Internet users [47-49]. We made an equal allocation to the eight sample groups categorized by sex and age to minimize selection bias; however, there was still an unavoidable bias in the representativeness of the participants registered with the Internet research company. Among the registered participants, approximately 50% were male, approximately 55% were in their twenties and thirties, and approximately 45% had graduated from college or graduate school. By contrast, in the general Japanese population, one national survey found that approximately 30% of people were in their twenties and thirties among adults older than 20 years, and approximately 20% of people had graduated from college or graduate school [50]. Moreover, previous studies have indicated that respondents may have certain characteristics, such as having higher income, frequent access to the Internet, and being more likely to respond to a survey than the general Internet user population [48,49]. Therefore, it is necessary to note that the 2115 participants in this study were younger, more educated, had a higher income, and had greater Internet access than population of Internet users and the general population in Japan.

Second, health behavior and eHealth literacy were assessed only using a self-administered questionnaire. Inaccuracies in estimating health behavior and eHealth literacy level were thus unavoidable. Moreover, some studies have reported that eHEALS is inappropriate because it does not assess the ability to use Web 2.0 [18,21]. Therefore, it is necessary to improve the model of eHealth literacy to fit the rapid changes in the informational landscape created by Web 2.0 tools [18].

### Conclusions

Among Japanese adult Internet users, we found some health behaviors, including exercise and balanced nutrition, to be independently associated with eHealth literacy. In rapidly developing Internet user societies, further research is needed to identify the mechanisms linking eHealth literacy with health information seeking and health behavior toward designing effective strategies more precisely for promoting healthy behavior.

## Appendix

Multimedia Appendix 1

## Abbreviations

AOR: adjusted odds ratio
eHEALS: eHealth Literacy Scale
IMeHU: Integrative Model of eHealth Use
