# Supplementary material for: Associations of eHealth Literacy With Health Behavior Among Adult Internet Users
Source: J Med Internet Res. 2016 Jul 18;18(7):e192. doi: 10.2196/jmir.5413 (PMC4969548; doi:10.2196/jmir.5413)
Supplement: Multimedia Appendix 1 [file jmir_v18i7e192_app1.pdf]

Multimedia Appendix 1: eHealth Literacy Scale [19]

I would like to ask you for your opinion and about your experience using the Internet for health information. For each statement, tell me which response best reflects your opinion and experience *right now*.

1. I know **what** health resources are available on the Internet

1) ❑ Strongly Disagree
2) ❑ Disagree
3) ❑ Undecided
4) ❑ Agree
5) ❑ Strongly Agree

2. I know **where** to find helpful health resources on the Internet

1) ❑ Strongly Disagree
2) ❑ Disagree
3) ❑ Undecided
4) ❑ Agree
5) ❑ Strongly Agree

3. I know **how** to find helpful health resources on the Internet

1) ❑ Strongly Disagree
2) ❑ Disagree
3) ❑ Undecided
4) ❑ Agree
5) ❑ Strongly Agree

4. I know **how to use** the Internet to answer my questions about health

1) ❑ Strongly Disagree
2) ❑ Disagree
3) ❑ Undecided
4) ❑ Agree
5) ❑ Strongly Agree

5. I know how to use **the health information** I find on the Internet to help me

1) ❑ Strongly Disagree
2) ❑ Disagree
3) ❑ Undecided
4) ❑ Agree
5) ❑ Strongly Agree

6. I have the skills I need to **evaluate** the health resources I find on the Internet

1) ❑ Strongly Disagree
2) ❑ Disagree
3) ❑ Undecided
4) ❑ Agree
5) ❑ Strongly Agree

7. I can tell **high quality** health resources from **low quality** health resources on the Internet

1) ❑ Strongly Disagree
2) ❑ Disagree
3) ❑ Undecided
4) ❑ Agree
5) ❑ Strongly Agree

8. I feel **confident** in using information from the Internet to make health decisions

1) ❑ Strongly Disagree
2) ❑ Disagree
3) ❑ Undecided
4) ❑ Agree
5) ❑ Strongly Agree

Thank you!
